# Sustained polymorphic ventricular tachycardia induced during radiofrequency catheter ablation of idiopathic right ventricular outflow tract extrasystoles

**Published:** 2011-03-25

**Authors:** Konstantinos P Letsas, Michael Efremidis, Dimitrios Bramos, Charis Charalampous, Spyros Tsikrikas, Paraskevi Skoufogianni, Antonios Sideris

**Affiliations:** Second Department of Cardiology, Division of Cardiac Electrophysiology, Evangelismos General Hospital of Athens, 10676 Athens, Greece

**Keywords:** polymorphic ventricular tachycardia, right ventricular outflow tract extrasystole, radiofrequency catheter ablation

## Case presentation

**Figure 1:**
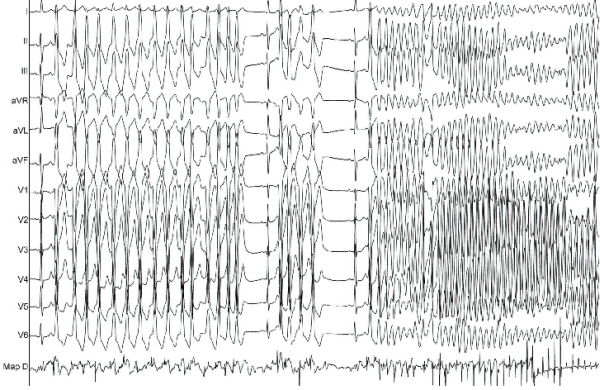
ECG tracing showing an episode of non-sustained monomorphic ventricular tachycardia and an episode of sustained polymorphic ventricular tachycardia (>30s) induced during radiofrequency catheter ablation.  Map D: distal bipole of the ablation catheter.

A 60-year-old male with a long-lasting history of palpitations was referred to our hospital for further evaluation. His family history was negative for sudden cardiac death at a young age. Transthoracic echocardiography and exercise treadmill test ruled out structural heart disease. Baseline ECG showed sinus rhythm and frequent premature ventricular contractions (PVCs) displaying rS pattern in leads V1 through V3, late transition zone in lead V4 and inferior axis. QTc interval (Bazzet's formula) was normal. Ambulatory ECG monitoring showed frequent monomorphic PVCs and bigeminy without episodes of ventricular tachycardia. After obtaining an informed consent, an electrophysiological study was carried out. A detailed activation mapping of the PVCs at the right ventricular outflow tract (RVOT) was performed with a 4-mm tip deflectable catheter (NAVISTAR, Biosense Webster) using a 3-dimentional electroanatomical mapping system (CARTO 3, Biosense Webster, Inc., Diamond Bar, CA, USA). The earliest activation site of the PVCs was identified at the anterior aspect of the RVOT free wall. Pace-mapping at this location demonstrated "perfect match" (12 of 12 leads) with the morphology of the PVCs. Shortly after the delivery of the radiofrequency (RF) energy, an episode of non-sustained monomorphic VT and an episode of sustained polymorphic VT (>30s) necessitating cessation of the ablation and external defibrillation were developed (Figure 1). Both episodes were initiated by PVCs of the same morphology with a relatively long coupling interval (416 ms for the monomorphic VT and 400 ms for the polymorphic VT). Additional RF energy applications at this area abolished all PVCs.

## Discussion

The large majority of idiopathic RVOT tachycardias are due to cyclic AMP-mediated triggered activity [1]. Application of RF energy in sinus rhythm at the target site may result in induction of repetitive PVCs or tachycardia with QRS characteristics similar to the PVCs [11]. A sudden increase in temperature of calcium overloaded cells has been shown to lead in the development of oscillatory afterdepolarizations, and subsequent triggered activity [2]. Hyperthermia may additionally cause membrane depolarization, reversible and irreversible loss of excitability, and abnormal automaticity [3]. These observations suggest that PVCs or tachycardias initiated during RF ablation may be due to thermal facilitation of triggered activity, but may also reflect thermally-induced abnormal automaticity. Sustained polymorphic VT/ventricular fibrillation during RF ablation of RVOT PVCs/tachycardia requiring external cardioversion have been rarely described [4,5]. Thermally-induced triggered activity and/or abnormal automaticity are the most plausible mechanisms. Other less possible underlying mechanisms include ischaemia and mechanical stimulation induced by the ablation catheter.

RVOT PVCs and/or tachycardia in subjects without structural heart disease are generally considered benign [1,6]. However, malignant forms of VT, ventricular fibrillation, and/or polymorphic VT are occasionally initiated by PVCs arising from the RVOT [6,7]. Functional block and/or delayed conduction by rapid firing due to triggered activity or microreentry arising from a single focus may lead to chaotic ventricular conduction causing ventricular fibrillation and/or polymorphic VT [6]. In our case, both episodes of monomorphic and polymorphic VT and were initiated by the target PVCs. Although, the coupling interval of the PVC was relatively long in both episodes, it was shorter in polymorphic VT. Viskin et al. described three patients with typical benign RVOT VT in whom malignant polymorphic VT developed during follow-up [7]. In this series, the coupling interval of the PVC initiating polymorphic VT was intermediate between the very short coupling interval initiating idiopathic VF and the long coupling interval inducing the truly benign RVOT VT [7]. Although, there are no specific markers to distinguish benign from malignant RVOT PVCs, a shorter coupling interval of the PVC, a longer QRS duration of the PVC as well as a shorter tachycardia cycle length are more commonly seen in the malignant form [6].
